# Urocortin 3 overexpression reduces ER stress and heat shock response in 3T3-L1 adipocytes

**DOI:** 10.1038/s41598-021-95175-4

**Published:** 2021-08-02

**Authors:** Sina Kavalakatt, Abdelkrim Khadir, Dhanya Madhu, Heikki A. Koistinen, Fahd Al-Mulla, Jaakko Tuomilehto, Jehad Abubaker, Ali Tiss

**Affiliations:** 1 Biochemistry and Molecular Biology Department, Research Division, Dasman Diabetes Institute, P.O. Box 1180, 15462 Dasman, Kuwait; 2 University of Helsinki and Department of Medicine, Helsinki University Central Hospital, Helsinki, Finland; 3 Minerva Foundation Institute for Medical Research, Helsinki, Finland; 4 Department of Public Health Solutions, Finnish Institute for Health and Welfare, Helsinki, Finland; 5 Research Division, Dasman Diabetes Institute, Dasman, Kuwait; 6 Department of Public Health, University of Helsinki, Helsinki, Finland

**Keywords:** Biochemistry, Diseases, Endocrinology

## Abstract

The neuropeptide urocortin 3 (UCN3) has a beneficial effect on metabolic disorders, such as obesity, diabetes, and cardiovascular disease. It has been reported that UCN3 regulates insulin secretion and is dysregulated with increasing severity of obesity and diabetes. However, its function in the adipose tissue is unclear. We investigated the overexpression of UCN3 in 3T3-L1 preadipocytes and differentiated adipocytes and its effects on heat shock response, ER stress, inflammatory markers, and glucose uptake in the presence of stress-inducing concentrations of palmitic acid (PA). UCN3 overexpression significantly downregulated heat shock proteins (HSP60, HSP72 and HSP90) and ER stress response markers (GRP78, PERK, ATF6, and IRE1α) and attenuated inflammation (TNFα) and apoptosis (CHOP). Moreover, enhanced glucose uptake was observed in both preadipocytes and mature adipocytes, which is associated with upregulated phosphorylation of AKT and ERK but reduced p-JNK. Moderate effects of UCN3 overexpression were also observed in the presence of 400 μM of PA, and macrophage conditioned medium dramatically decreased the UCN3 mRNA levels in differentiated 3T3-L1 cells. In conclusion, the beneficial effects of UCN3 in adipocytes are reflected, at least partially, by the improvement in cellular stress response and glucose uptake and attenuation of inflammation and apoptosis.

## Introduction

The corticotropin-releasing factor (CRF) family of neuropeptides and receptors is implicated in the suppression of food intake and inflammation as well as in the regulation of energy balance^1^. The CRF family consists of ligands, CRF, CRF-bp, and urocortins (UCN) 1,2,3, with their receptors, CRHR1 and CRHR2. These neuropeptides are expressed in discrete areas of the brain and in the adipose tissue, pancreas, skeletal muscle, and heart^2–4^. They may play a significant role in various endogenous pathways, including stress adaptation^1^. For instance, the administration of UCN1 exerts cardioprotective effects on cardiomyocytes^5^. The activation of CRFR2 by UCN2 and UCN3 also activates the *Ras* and *Raf*-1 kinase pathways that are critical for cardioprotection^5^. UCNs are affected by environmental challenges, such as oxidative stress^6,7^ and alcohol consumption^8^. Interestingly, the disruption of CRF2 signaling causes distortion of the endoplasmic reticulum (ER) morphology, accompanied by ER stress signaling. Uncontrolled protein ubiquitination affects protein synthesis, secretion, and mistargeting under this condition^9^.

UCN3 is a recently characterized CRF family member and binds specifically to receptor CRHR2^10,11^. UCN3 and CRHR2 are highly expressed in pancreatic β cells, where UCN3 modulates insulin secretion through the regulation of somatostatin secretion^4^. Moreover, they are co-expressed in the adipose tissue and adipocytes^12^, suggesting local activity in this tissue. UCN3 is involved in energy homeostasis^13,14^, and mice with global UCN3 overexpression exhibit resistance to metabolic impairment that is related to a high-fat diet^2^. The local overexpression of UCN3 in the muscle improves local glucose homeostasis^2^. Furthermore, impaired UCN3 expression is reported in various metabolic syndromes, such as type 2 diabetes (T2D), obesity, polycystic ovary syndrome (PCOS), and sleep apnea^4,12,15^. Interestingly, UCN3 and CRHR2 are proposed as potential anti-obesity targets owing to their co-location with quantitative trait loci for obesity on chromosome 10p15.1^16^. Recently, we reported that the plasma UCN3 levels decrease with increased body weight in human subjects without T2D; however, such levels increase with concomitant T2D^12^. Conversely, the UCN3 levels in the same subjects in the subcutaneous adipose tissue (SAT) increase with body weight and decrease with T2D^12^. These findings indicate the complex and crucial roles of UCN3 as a marker/target for controlling metabolic disease progression.

In obese individuals, the adipose tissue is infiltrated with macrophages and is characterized by chronic metabolic stress and low-grade inflammation, which are reflected in disrupted cellular host defense mechanisms^17^. In addition, impaired defense mechanism and ER stress response are observed in obesity and diabetes^18^. This impairment includes also heat shock response (HSR) considered as a second cellular defense response—critical for protein refolding, repair, and cellular homeostasis—in individuals with obesity^19^ and diabetes^20^. Some HSR proteins (HSPs) are ubiquitously expressed, whereas others are expressed under stress conditions. HSP27 inhibits IKKβ and regulates TNFα-induced NF-κB activation^21^. HSP72 activation is protective through the binding and repression of JNK phosphorylation^22,23^. In our previous work, we investigated the impairment of HSPs regulation in obesity and T2D^24,25^. HSR may play a significant role in endoplasmic reticulum quality control and could relieve ER stress through various pathways^26^. GRP78 protein, a HSP and master regulator for ER stress canonical markers, is key to crosstalk between HSR and ER stress responses^27^. Three ER resident proteins, protein kinase RNA-like endoplasmic reticulum kinase (PERK), activating transcription factor-6 (ATF6), and inositol-requiring enzyme-1α (IRE1α) are primarily responsible for sensing ER perturbations caused by physiological changes in extracellular conditions^28,29^.

We hypothesized that the potential protective role of UCN3 in obesity and diabetes is exercised at least partially by facilitating cellular stress response in the adipose tissue. We overexpressed UCN3 in 3T3-L1 adipocytes and treated them with palmitic acid (PA) to induce stress. Then, we investigated the status of HSR and ER stress response, glucose uptake, and inflammation.

## Results

### UCN3 overexpression enhances cellular stress response under palmitic acid stress

**Figure 1:**
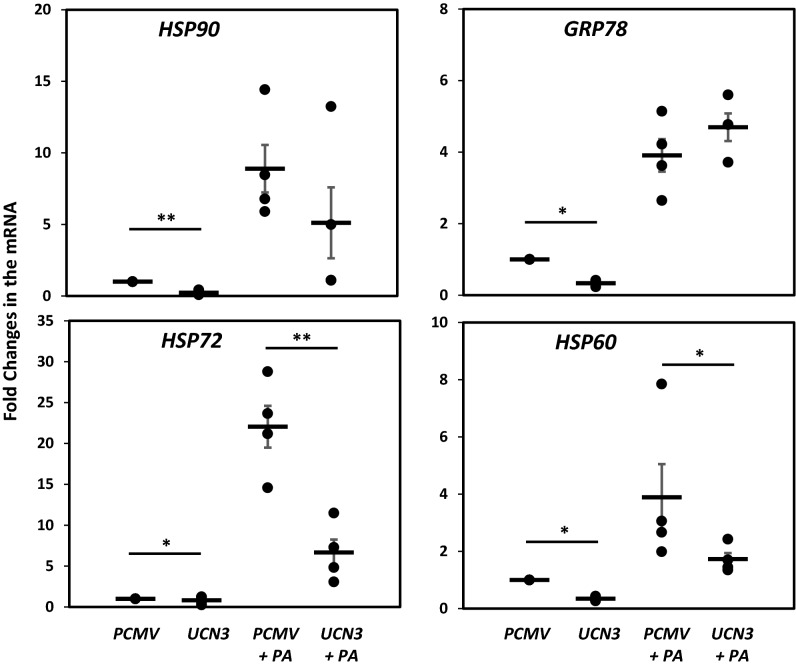
Expression profile of HSPs in 3T3-L1 adipocytes under PA treatment. mRNA expression levels of HSP60, HSP72, HSP90, and GRP78 were measured by quantitative real-time PCR in 3T3-L1 adipocytes in the absence and presence of PA (400 µM) overnight. GAPDH was used as an internal control for normalization and data are presented as fold changes under each condition compared with adipocytes transfected with PCMV from independent experiments (n = 4). *p < 0.05; **p < 0.01.

**Figure 2:**
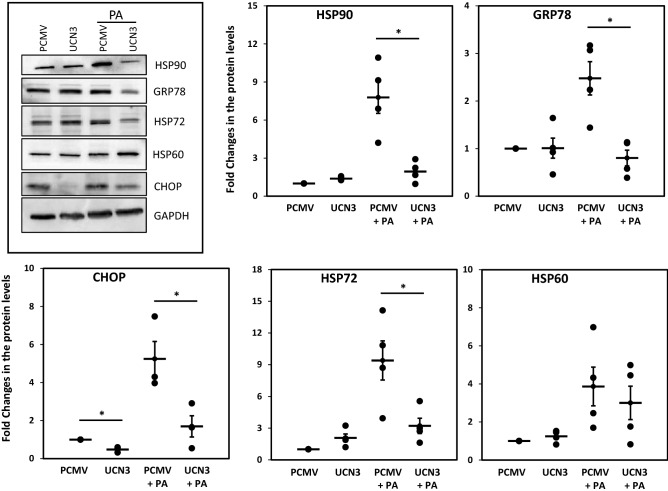
Expression levels of HSPs and CHOP in 3T3-L1 adipocytes under PA treatment. The protein levels were measured by Western Blot using 3T3-L1 adipocytes treated overnight with PA (400 µM). Gels are representative of at least three independent experiments. Quantitative data are normalized to internal GAPDH and presented as fold changes under each condition compared with adipocytes transfected with PCMV (n = 4). *p < 0.05.

To elucidate the potential protective role of UCN3 in metabolic disorders, such as obesity and diabetes, UCN3 was overexpressed in 3T3-L1 differentiated adipocytes without and with stress-inducing amounts of PA (400 μM) as shown in Figure S1. Overexpression of UCN3 in the absence of PA significantly downregulated the expression of heat shock proteins, HSP60, HSP72, HSP90, and GRP78, at the mRNA expression levels (*p* < 0.05) (Fig. 1). Treatment with PA significantly increased (*p* < 0.01) the mRNA expression levels of these proteins. However, the increase of HPS60 and HSP72 (*p* < 0.05 and *p* < 0.01, respectively) was attenuated by UCN3 overexpression. We then evaluated the effects of UCN3 overexpression at the protein levels via western blotting. Despite the more pronounced downregulation of HSP90, GRP78 and HSP72 levels due to this overexpression observed in the presence of PA, HSP60 protein levels were not decreased as shown above at mRNA levels (Fig. 2). Moreover, only a marginal effect of UCN3 overexpression was noted in the absence of PA in contrast to the significant decrease in the mRNA levels of all HSPs shown in Fig. 1.

**Figure 3:**
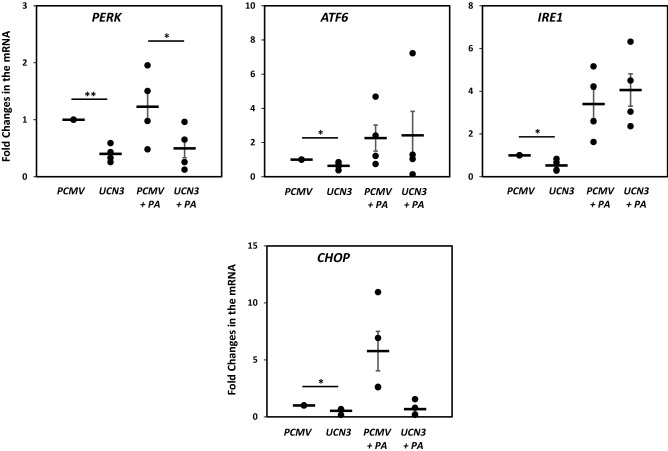
Expression profile of ER stress markers in 3T3-L1 adipocytes under PA treatment. mRNA expression levels of PERK, ATF6, IRE1α, and CHOP were measured by quantitative real-time PCR in 3T3-L1 adipocytes in the absence and presence of PA (400 µM) overnight. GAPDH was used as an internal control for normalization and data are presented as fold changes under each condition compared with adipocytes transfected with PCMV from independent experiments (n = 4). *p < 0.05; **p < 0.01.

We further evaluated the impact of UCN3 overexpression on ER stress markers and the apoptotic marker CHOP. Interestingly, the CHOP expression levels were significantly downregulated at both the mRNA and protein levels in the presence and absence of PA (Figs. 2 and 3). UCN3 overexpression significantly downregulated the PERK mRNA expression levels in the presence and absence of PA (*p* < 0.05), although this attenuation was observed only in the absence of PA for other ER stress regulators, ATF6 and IRE1α (Fig. 3), and their downstream genes eIF2α and XBP1 (data not shown).

### UCN3 overexpression increases glucose uptake

**Figure 4:**
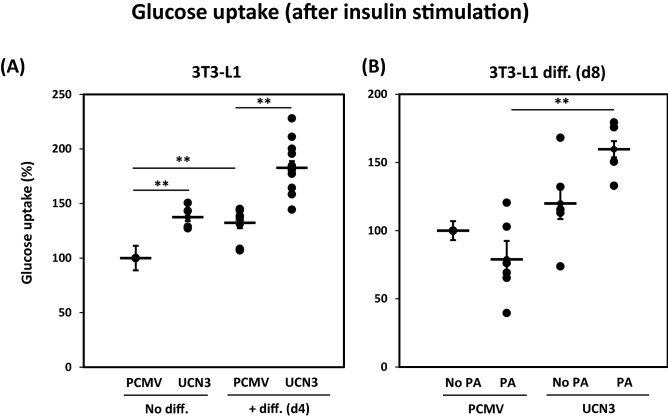
Glucose uptake in 3T3-L1 preadipocyte and differentiated adipocyte cells. Glucose uptake experiments were measured as detailed in material and methods. Data are presented as percentage increase of glucose uptake under each condition compared with cells transfected with PCMV in absence of PA from independent experiments (n = 7 to 9). **p < 0.01.

We evaluated glucose uptake in 3T3-L1 adipocytes in the presence or absence of PA treatment, under insulin-stimulated conditions, to further investigate the positive effect of UCN3 overexpression. As shown in Fig. 4A, glucose uptake was significantly increased in preadipocytes transfected with UCN3 in comparison with the preadipocytes transfected with PCMV control (*p* < 0.01). This increase in glucose uptake was further enhanced (*p* < 0.01) after continuing 3T3-L1 cell differentiation up to 4 days with ectopic UCN3 overexpression. This enhancement was observed despite a part of the increase being caused by differentiation (Fig. 4A). Comparable but lesser effects were observed when UCN3 was overexpressed in mature differentiated 3T3-L1 cells (day 8) (*p* < 0.05, Fig. 4B). Comparable effects of UCN3 overexpression on glucose uptake were also observed in basal conditions (without insulin stimulated) as shown in Figure S2.

**Figure 5:**
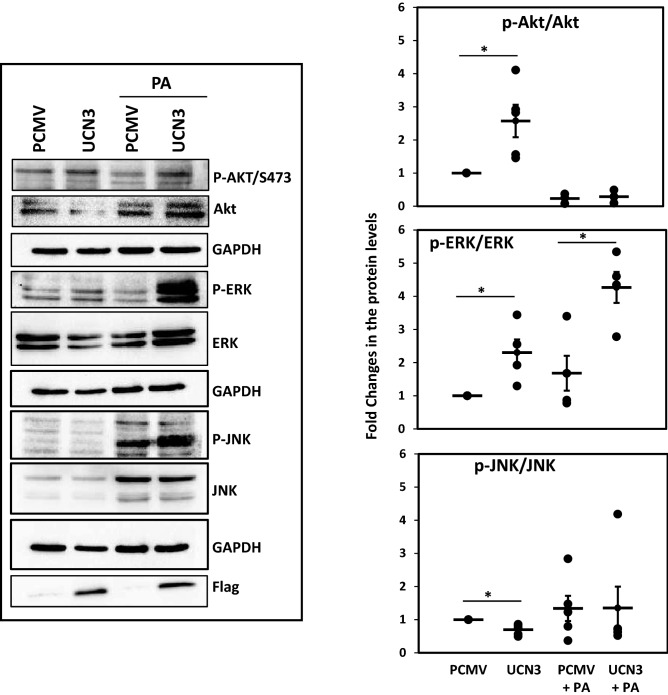
Expression and phosphorylation levels of Akt, JNK and ERK proteins in 3T3-L1 adipocytes under PA treatment and basal insulin conditions. The protein levels were measured by Western blot using 3T3-L1 adipocytes treated overnight with PA (400 µM). Gels are representative of at least three independent experiments. Quantitative data are normalized to internal GAPDH and presented as fold changes under each condition compared with adipocytes transfected with PCMV (n = 5). *p < 0.05.

We then investigated the effect of UCN3 overexpression on insulin signaling effectors to provide a preliminary explanation to such effects. We therefore examined the expression and phosphorylation levels of Akt, ERK, and JNK proteins via western blotting. UCN3 overexpression significantly increased phosphorylation of both Akt/S473 and ERK and decreased phosphorylation of JNK under basal conditions of insulin (*p* < 0.05, Figure S3 and 5). After insulin stimulation, comparable trends were observed but less pronounced for p-Akt and p-ERK. We further assessed the effect of UCN3 overexpression on these markers in the presence of PA, under basal insulin conditions. While UCN3 increased the phosphorylation of Akt and ERK in the absence of PA, in its presence, however, only p-ERK was significantly increased (*p* < 0.05, Fig. 5).

### Urocortin 3 overexpression decreases inflammation

**Figure 6:**
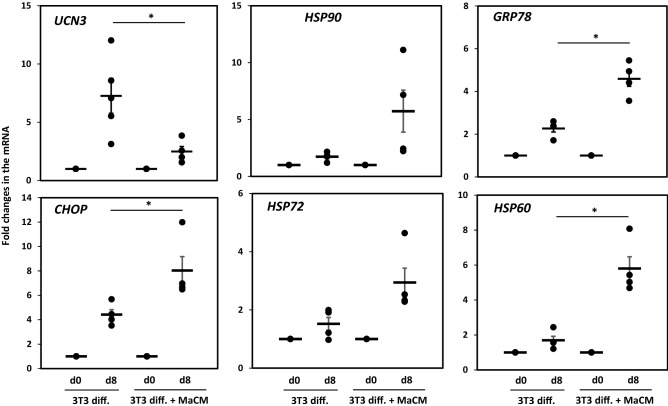
Expression levels of UCN3 and HSPs during the differentiation of 3T3-L1 preadipocytes and their treatment with a macrophage culture medium (MaCM). mRNA expression levels of UCN3, HSP90, GRP78, CHOP, HSP72, and HSP60 were measured by quantitative real-time PCR in 3T3-L1 preadipocytes (d0) and adipocytes differentiated for 8 days (d8) with and without MaCM. GAPDH was used as an internal control for normalization and data are presented as fold changes in differentiated adipocytes compared with preadipocytes from independent experiments (n = 4). *p < 0.05.

**Figure 7:**
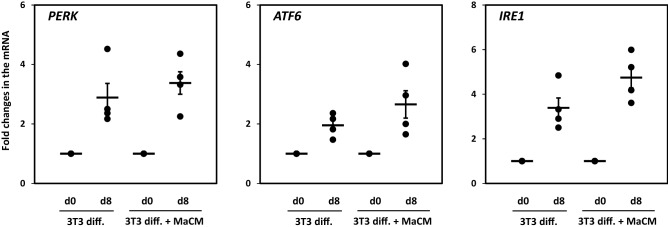
Expression levels of ER stress and apoptotic markers during the differentiation of 3T3-L1 preadipocytes and their treatment with a macrophage culture medium (MaCM). mRNA expression levels of PERK, ATF6, and IRE1α were measured by quantitative real-time PCR in 3T3-L1 preadipocytes (d0) and adipocytes differentiated for 8 days (d8) with and without MaCM. GAPDH was used as an internal control for normalization and data are presented as fold changes in differentiated adipocytes compared with preadipocytes from independent experiments (n = 4). *p < 0.05.

Low-grade chronic inflammation is a characteristic of adipose tissue in patients with obesity and insulin resistance-resistant patients^30^. 3T3-L1 preadipocytes were differentiated in the presence of RAW264.7 macrophage conditioned medium (MaCM) using an indirect co-culture method to mimic inflammatory conditions. We evaluated the expression levels of UCN3 and HSPs. Under normal conditions, UCN3 mRNA expression was increased with differentiation, as we previously reported^12^. However, a clear downregulation of the UCN3 mRNA levels (*p* < 0.05) was observed in the presence of MaCM (Fig. 6). Under MaCM stress-inducing conditions, the expression levels of HSP60, HSP72, HSP90, and GRP78 were upregulated, as expected. A trend of increased expression of ER stress markers, PERK, ATF6, and IRE1α, was also observed; however, it did not reach statistical significance (Fig. 7). The effect was more pronounced for the apoptotic marker, CHOP (*p* < 0.05).

These observations prompted us to investigate the effect of UCN3 overexpression on representative inflammatory markers. Overexpression significantly decreased the expression levels of TNFα mRNA in the absence and presence of PA (Figure S6). Moreover, a similar decrease in the IL6 mRNA expression levels was observed in the presence of PA, but the opposite effect was observed in the absence of PA (Figure S6).

### Urocortin 3 is negatively associated with circulating HSP60 and GRP78 in adult humans with obesity

In a previous study, we reported a decrease in UCN3 levels in the plasma of subjects that are overweight and subjects with obesity compared with normal-weight individuals^12^. Based on the effects of UCN3 overexpression on HSPs and ER markers in adipocytes, and the opposite trends in the UCN3 and HSPs levels in MaCM, we revisited our previous data and conducted correlation analyses to evaluate the circulating levels of UCN3, HSP60, and GRP78. A description of the population study, the methods used, and the results are detailed in previous reports^12,24,31^. Interestingly, significant negative correlations between the UCN3 levels and the HSP60 and GRP78 levels were observed (R =  − 0.384, *p* = 0.001, and R =  − 0.285, *p* = 0.006, respectively) using data from 144 non-diabetic humans with various body mass indices (Figure S7). However, no correlation was observed in the analysis of similar data for a subgroup with diabetes (data not shown).

## Discussion

UCN3 exhibits a possible beneficial activity for metabolic disorders, such as obesity and diabetes. Moreover, it is dysregulated with increasing severity in patients with obesity and diabetes^12^. However, its function in the adipose tissue is unclear. In a previous study^12^, we reported the status of UCN3 in the plasma and adipose tissue of subjects with overweight or obesity. In the present study, we demonstrate that UCN3 overexpression in 3T3-L1 adipocytes reduces heat shock and ER stress responses, and attenuates inflammation and apoptosis. These events are associated with improved glucose uptake in both pre-mature and mature adipocytes. Comparable effects are also observed in the presence of PA as a metabolic stressor. We found negative correlations between the circulating levels of UCN3 and HSP60 as well as GRP78 in human plasma and in MaCM differentiated 3T3-L1 adipocytes. To the best of our knowledge, this report is the first to elucidate the mechanisms for the protective effect of UCN3 overexpression in adipocytes accompanied by enhanced glucose uptake and insulin signaling through the upregulation of phosphorylation of Akt and ERK but downregulation of p-JNK.

UCN3 and its receptor CRHR2 are expressed in various tissues, including the pancreatic β-cells and adipose tissue^3,4^. Co-expression of UCNs and their receptors within the same tissues may indicate a physiologic paracrine function. In support of this, UCN3 overexpression improves glucose uptake and insulin signaling in the skeletal muscle through an autocrine/paracrine mechanism with upregulated GLUT1,4 and AMPK^2^. Moreover, UCN3 gene transfer exerts beneficial effects on weight loss, reduced liver fat, and enhanced glucose control^32^. We also reported previously that the levels of UCN3 in SAT were increased in obesity but attenuated in diabetes, which is consistent with the low UCN3 expression in the β-cells obtained from subjects with diabetes^12^.

Interestingly, our results demonstrated increased glucose uptake both by preadipocytes and mature adipocytes when UCN3 is overexpressed. In agreement with these results, UCN3 + transgenic mice are likely protected from metabolic challenges by a favorable phenotype that counters obesity and hyperglycemia, possibly via augmented glucose metabolism and uptake as well as fatty acid metabolism^33^. Furthermore, UCN3 KO mice exhibited improved insulin sensitivity and metabolic resiliency under high-fat feeding, whereas aged UCN3 KO mice exhibited better glucose homeostasis compared with WT mice^34,35^. By disturbing the UCN3 levels, both UCN3 overexpression and UCN3 KO mice exhibited metabolic protection. Moreover, muscle CRFR2 stimulation improved muscle mass and glucose homeostasis^33,36^. Hence, the improved metabolic status observed is at least partially due to UCN3 overexpression and action in the periphery.

Conversely, HSPs are key players in mitigating cellular and tissue stress to maintain cellular homeostasis through crosstalk with ER stress and inflammation regulators^17,19^. In humans with diabetes, the levels of HSPs, in particular HSP72, decreased^20^, but in subjects with obesity without diabetes, we recently observed increased levels in both the adipose tissue and blood^24,25,31^. These contrasting findings in two metabolic disorders, which are known to be associated with insulin resistance, indicate that HSR might resolve moderate metabolic stress related to obesity, but not more severe stress observed in diabetes. Our current results further support this hypothesis and provide additional evidence on the involvement of HSPs in cellular resilience under stress. Exposure to high concentrations of PA increased HSR, ER stress response markers, and apoptosis marker, CHOP, as expected. However, UCN3 overexpression before PA treatment decreased the level of HSPs in adipocytes and likely eliminated the requirement for the activation of these defense mechanisms. Of note, the variations in the HSP gene and protein expression levels upon the UCN3 overexpression were not always correlating. This could be due to multiple posttranslational modifications or change in the cell environment modulating its protein levels under metabolic stress conditions. The complex crosstalk and back-regulation between the HSR, ER stress markers, and inflammation might also impact the need for translation of HSPs. This may particularly affect GRP78 and HSP60 involved in the UPR system and inflammatory reaction, respectively.

Notably, UCN3 overexpression had a limited impact on ER stress markers in the presence of PA compared with its absence. Similar trends are also observed in phosphorylation of Akt, JNK, and ERK, which are all involved in insulin signaling. This may be explained by an overwhelming exposure to stress that exceeds the cellular response capacity of these sensitive and canonical regulators and thus impairs their functions in metabolic diseases, such as diabetes. We and others previously reported that the expression levels of ER stress master regulator GRP78 are differentially dysregulated in obesity and diabetes^24,25,31^. UCN3 may mitigate metabolic events through enhanced cellular stress defense mechanisms. The clear downregulation in the CHOP levels observed in the absence and presence of PA associated with UCN3 overexpression is consistent with the previous studies.

Numerous metabolic disorders are also associated with inflammation, mitochondrial dysfunction, and apoptosis^37^. It has been reported that UCNs exert anti-inflammatory activity via the PI3K/Akt and GSK pathways^38^. Further, the cardioprotective effect of UCN1, UCN2, and UCN3 is suggested to involve the MAPK pathway^40–41^. We observed UCN3 anti-inflammatory activity in the presence of PA, as reflected in the downregulation of the TNFα and IL6 expression levels. However, the increase in IL6 expression due to UCN3 overexpression in the absence of PA is unexpected. Aside from its established pro-inflammatory role, IL6 cytokine also exhibits numerous non-inflammatory physiological actions, including increased glucose disposal, lipolysis, glucose and increased energy expenditure^42^. When activation is prolonged, IL6 becomes pathogenic; however, short-term IL6 signaling exerts cardioprotective effects on myocardial infarction in response to tissue damage^43^. Thus, the attenuation of some cellular defense systems, such as HSR, due to UCN3 overexpression may be compensated by the increased levels of IL6 to fulfill some physiological roles.

We also observed a significant negative correlation between the UCN3 level and HSP60 and GRP78 levels in human plasma. However, this association is not observed in subjects with diabetes. Moreover, in a previous report^12^, we observed an association between UCN3 and the regulated on activation, normal T cell expressed and secreted chemokine (RANTES) in subjects with diabetes, which was not present in the non-diabetic patients. Consistent with these findings, we report that indirect co-culture with MaCM induces a decrease in UCN3 expression and an increase in ER stress, HSPs, and CHOP levels. Conditioned medium represents low-grade inflammation in the adipose tissue, as observed in obesity. Hence, the local microenvironment may affect the expression levels of UCN3, and UCN3 may participate in adaptive responses to maintain homeostasis. However, with chronic stress, this effect is attenuated.

Our findings are in agreement with those of previous studies, which demonstrate that increased UCN3 levels may exert protective effects against moderate or transient metabolic insults. Still, the physiological role and mechanism of action of UCN3 are complex, and its beneficial effects require signal integration among cell types and crosstalk among numerous adaptive mechanisms, both centrally and peripherally. Hence, a better understanding of the molecular mechanisms that regulate UCN3 expression and action is expected to provide critical insight into its role in the pathophysiology of various metabolic disorders.

To the best of our knowledge, the present study is the first to report enhanced heat shock and ER stress responses and glucose uptake with UCN3 overexpression in adipocytes under metabolic stress. In vitro work is supported with data from adult human plasma. However, despite our novel findings, this study has some limitations, such as lack of knockdown experiments for the validation and assessment of the status of the CRHR2 receptor in used cells. A detailed characterization of insulin signaling pathways under metabolic stress following UCN3 overexpression is planned, and the results are expected to further elucidate the action of UCN3 on adipocytes in the context of obesity and diabetes.

## Methods

### Cell culture, transfection, and treatment

Mouse preadipocyte (3T3-L1) and macrophage (RAW264.7) cells were purchased from the American Type Culture Collection (VA, USA) and then cultured as previously described^12,44^. Briefly, 3T3-L1 cells were normally differentiated into mature adipocytes until day 8, and in indirect co-culture, 3T3-L1 cell differentiation was induced in conditioned media from RAW264.7 cells. Oil red O staining of lipid droplets was used to monitor cell differentiation as previously described^12^.

Human UCN3 gene cloned in pCMV6 vector tagged with Myc-DDK (OriGene, Rockville, MD, USA) was used for transfection. Myc-DDK tagged pCMV6 vector without UCN3 (OriGene, Rockville, MD, USA) was used as a control. 3T3-L1 adipocytes were transfected via electroporation using the Cell Line Nucleofector Kit (Lonza, Basel, Switzerland) according to the manufacturer’s instructions. Briefly, 2 × 10^6^ cells were electroporated with 2 μg of plasmid using Nucleofector program, A-033. About 24 h after transfection, the cells were treated with 400 µm of PA (Sigma-Aldrich, St. Louis, MA, USA) in low glucose (1 g/l) and 1% (w/v) serum medium for 24 h with and without insulin (20 nM for 10 min). PA was prepared in 10% (w/v) fatty acid free bovine serum albumin (BSA). The cells were then processed for RNA and protein analyses or plated for glucose uptake assay.

### Glucose (2-NBDG) uptake assay

Adipocytes were placed into a 96-well plate for glucose uptake assay using a cell-based assay kit (#600470, Cayman Chemical, Ann Arbor, MI, USA) according to the manufacturer’s instructions. The culture medium was replaced with 100 µL of glucose-free medium 24 h after plating to starve cells. After 1 h of starvation, the medium was replaced with fluorescently labeled deoxy-d-glucose analog (2-NBDG) (150 µg/mL) and with and without insulin (0.1 µm) at 37 °C for 1 h. The cells were then washed with the buffer provided, and fluorescence was measured using a Synergy H4 plate reader (BioTek, Winooski, VT, USA) at 485/535 nm. Glucose uptake experiments were conducted on both preadipocytes and differentiated adipocytes transfected with UCN3 and pCMV6 plasmid. Experiments and measurements were also conducted on transfected preadipocytes differentiated until day 3.

### Quantitative real-time PCR

TRIzol reagent was used for total RNA extraction according to the manufacturer’s instructions. Isolated RNA was quantified using an Epoch spectrophotometer (BioTek, Winooski, VT, USA) prior to reverse transcription using a High-Capacity cDNA Reverse Transcription Kit (Applied Biosystems, Foster City, CA, USA). cDNA was then amplified with TaqMan gene expression assay or custom primers (Table S1) and normalized to glyceraldehyde 3-phosphate dehydrogenase (GAPDH) using Applied Biosystem 7500. In the gene expression quantitation, the ΔΔCT method was used and results were reported as fold change.

### Western blot analysis

Whole protein extracts from cells were prepared in RIPA buffer (50 mM Tris–HCl, pH 7.5, 150 mM NaCl, 1% (w/v) Triton X-100, 1 mM EDTA, 0.5% sodium deoxycholate, and 0.1% (w/v) SDS). Protein concentrations were determined using the Bradford method and normalized to β-globulin. Samples (20 µg) were prepared in loading buffer containing β-mercaptoethanol and resolved on 10% (w/v) SDS-PAGE gels. Then, proteins were transferred onto PVDF membranes (100 V for 75 min) and blocked for 2 h at room temperature (RT) using 5% (w/v) nonfat dried milk in Tris-buffered saline containing 0.05% Tween-20. Membranes were probed with the primary antibodies at 4 °C overnight [anti-UCN3 (bs-2786R, Bioss Antibodies Inc., MA, USA), anti-HSP90 (ADI-SPA-830-F, Enzo, Farmingdale, NY, USA), anti-HSP72 (ADI-SPA-810-F, Enzo, Farmingdale, NY, USA), anti-HSP60 (ADI-SPA-805-F, Enzo, Farmingdale, NY, USA), anti-GRP78 (ab32618, Abcam), anti-CHOP (2895S, Cell Signaling Technology, Danvers, MA, USA), p-AKT/AKT, p-JNK/JNK, p-ERK/ERK (9271, 9272, 4668s, CST 9252L, 9101, and 9102, respectively; Cell signaling, Danvers, MA, USA), GLUT-1 (cs-129395,cell signaling), GLUT-4 (cs-2231 s, cell signaling) and anti-GAPDH (ab2302, Millipore, Temecula, CA)]. Membranes were then washed and incubated with rabbit horseradish peroxidase (HRP)-conjugated secondary antibody (1:10,000 dilution) for 2 h at RT. Detection was performed using super sensitivity West Femto ECL reagent (Thermo Scientific, Waltham, MA, USA), protein bands were visualized by chemiluminescence, gel images were captured using the VersaDoc 5000 system (Bio-Rad, Hercules, CA, USA), and band intensities were measured using the Quantity One software (Bio-Rad, Hercules, CA, USA). GAPDH was used as an internal control for protein loading.

### Study population and Blood biochemistry analysis (previously detailed in^12,24,31^)

A total of 144 non-diabetic normal weight and overweight subjects (n = 37 and 107 resp.) and 98 T2D subjects were recruited for our study as previously reported^12^. Participants with prior history of major illness, use of supplements or medications affecting body composition or bone mass and those who performed any physical exercise within last 6 months before enrollment to study were excluded. The physical, clinical and biochemical characteristics have been previously reported. All subjects had provided informed written consent approved by ethics committee of Dasman Diabetes institute, in line with the principles of the Declaration of Helsinki.

Physical-clinical measurements including body weight, systolic and diastolic blood pressure were made. Peripheral blood samples were obtained, and plasma was collected to analyze various inflammatory and metabolic markers. Glucose and lipid levels were measured using Siemens Dimension Chemistry Analyzer (Diamond Diagnostics, Holliston, MA, USA). Haemoglobin A1c (HbA1c) levels were determined with the Variant™ device (Bio-Rad, Hercules, CA, USA). Levels of plasma UCN3 (#LS-F12902, Lifespan Biosciences, USA), HSP60 (ADI-EKS-600, Enzo, PA, USA), and GRP78 (#ADI-900-214, Enzo LifeSciences, Switzerland) were assayed using ELISA kits, according to manufacturer's instructions. Absorbances were measured on H4 Synergy plate reader (Biotek, Winooski, VT, USA).

### Statistical analysis

Statistical analyses were conducted using the SPSS software v25.0 (IBM SPSS Statistics for Windows, IBM Corp. Armonk, NY, USA). All descriptive statistics in the study are expressed as mean ± standard error and at least 3 repeats were performed for each experimental condition. Nonparametric Mann–Whitney U tests were employed to evaluate the differences in the means between pairs of cell conditions. Correlations among variables were estimated using Spearman’s correlation coefficient. All *p*-values < 0.05 were considered statistically significant.

## Supplementary Information

Supplementary Information.

## Data Availability

All generated data and resources are reported in this manuscript and there is no other data to be provided.

## Abbreviations

ATF6: Activating transcription factor 6
CHOP: C/EBP homologous protein
CRF: Corticotropin-releasing factor
CRHR: Corticotropin-releasing hormone receptor
ERK: Extracellular signal-regulated kinase
GAPDH: Glyceraldehyde 3-phosphate dehydrogenase
GRP78: 78-KDa glucose-regulated protein
HSP60: Heat shock protein 60
HSP72: Heat shock protein 72
HSP90: Heat shock protein 90
HSR: Heat shock response
IL6: Interleukin 6
IRE1α: Inositol-requiring enzyme-1α
JNK: C-Jun N-terminal kinase
MaCM: Macrophage conditioned medium
PA: Palmitic acid
PERK: Protein kinase RNA-like endoplasmic reticulum kinase
T2D: Type 2 diabetes
TNFα: Tumor necrosis factor α
UCN: Urocortin
UCN3: Urocortin 3
