# Supplementary material for: Urocortin 3 overexpression reduces ER stress and heat shock response in 3T3-L1 adipocytes
Source: Sci Rep. 2021 Aug 2;11:15666. doi: 10.1038/s41598-021-95175-4 (PMC8329193; doi:10.1038/s41598-021-95175-4)
Supplement: Supplementary file 1 — Supplementary Information. [file 41598_2021_95175_MOESM1_ESM.pdf]

Urocortin 3 overexpression reduces ER stress and heat shock response in 3T3-L1 adipocytes

Sina Kavalakatt[1], Abdelkrim Khadir[1], Dhanya Madhu[1], Heikki A. Koistinen[2, 3, 4], Fahd Al-Mulla[5], Jaakko Tuomilehto[4, 6], Jehad Abubaker[1], and Ali Tiss[1]*

[1] Biochemistry and Molecular Biology department, Research Division, Dasman Diabetes Institute, Kuwait

[2] University of Helsinki and Department of Medicine, Helsinki University Central Hospital, Helsinki, Finland

[3] Minerva Foundation Institute for Medical Research, Helsinki, Finland

[4] Department of Public Health Solutions, Finnish Institute for Health and Welfare, Helsinki, Finland

[5] Research Division, Dasman Diabetes Institute, Kuwait

[6] Department of Public Health, University of Helsinki, Helsinki, Finland

Supplementary Materials

Table S1: List of mouse primers used for RT-PCR Gene analysis

Gene	*Catalogue # or sequence*
UCN3	*Mm00453206 (Applied Biosystem, Waltham, MA, USA)*
GAPDH	*Mm99999915 (Applied Biosystem, Waltham, MA, USA)*
TNF	*Mm00443258 (Applied Biosystem, Waltham, MA, USA)*
IL6	*Mm00446190 (Applied Biosystem, Waltham, MA, USA)*
HSP90	*5′-GGCATCGATGAAGATGAGG-3′*<br>*5′-ACATGAGCAGAGAGCCAGGT-3′*
HSP72	*5′-TGCTGATCCAGGTGTACGAG-3'*<br>*5′-CGTTGGTGATGGTGATCTTG-3'*
HSP60	*5′-CGTTGCCAATAACACAAACG-3′*<br>*5′-CTTCAGGGGTTGTCACAGGT-3′*
GRP78	*5′-GGATGCGGACATTGAAGACT-3'*<br>*5′-TCCCAACGAAAGTTCCTGAG-3'*
CHOP	*5′-CGGAACCTGAGGAGAGAGTG-3'*<br>*5′-TATAGGTGCCCCCAATTTCA-3′*
ATF6	*5′-GGTTCAGCCCTGGACAAATA-3′*<br>*5′-CTGCTGATTAGCCGAGTTCC-3′*
PERK	*5′-GCCCAAACATCGAGAAAATG-3′*<br>*5′-GCTCCCAGCTTCTGCTTAGA-3'*
IRE1	*5′-GGCCACTTTGAACTTCGGTA-3′*<br>*5′-CCAATCAGCAACGGAAACTT-3′*

Figure S1. UCN3 overexpression levels in 3T3-L1 adipocyte cells in the absence of insulin stimulation (at basal level). The expression levels were measured by Western Blots and by RT- PCR using 3T3-L1 adipocytes transfected with UCN3 and PCMV plasmid vectors. Quantitative data are normalized to internal GAPDH and presented as fold changes compared with adipocytes transfected with PCMV (n=3 and n=3 to 6, for Western blots and RT-PCR, respectively). * $p < 0.05$, ** $p < 0.01$.

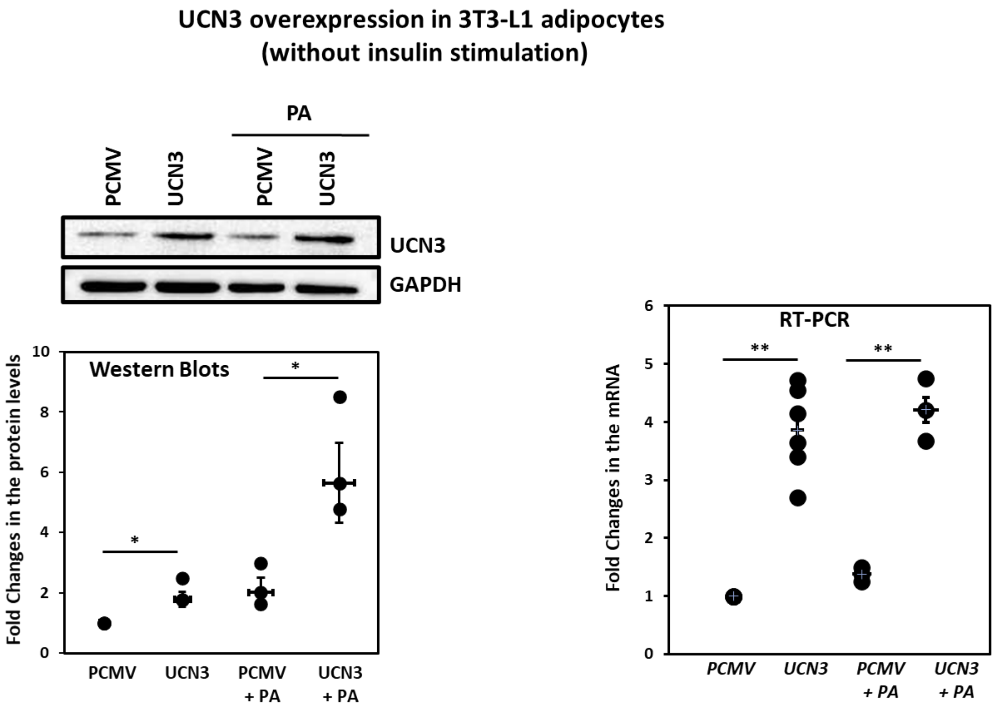

Figure S1

Figure S2. Glucose uptake in 3T3-L1 preadipocytes (A) and differentiated adipocyte (B) cells at basal level. Glucose uptake experiments were measured as detailed in material and methods. Data are presented as percentage increase of glucose uptake under each condition compared with cells transfected with PCMV in absence of PA (n=7 to 9, * *p* < 0.05, ** p < 0.01.

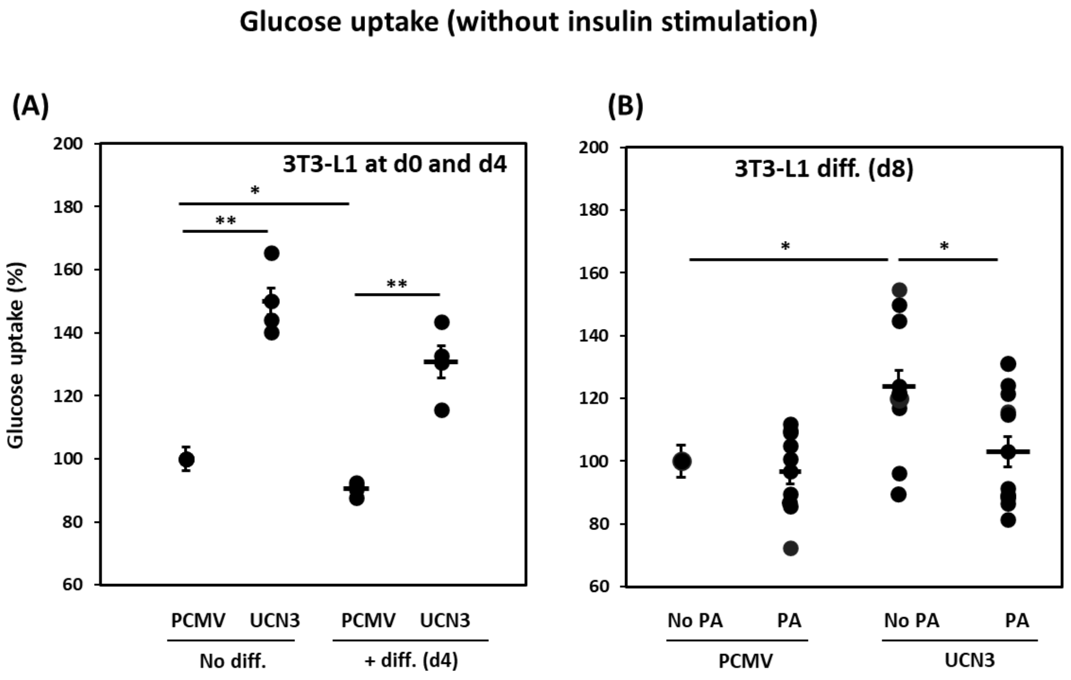

Figure S2

Figure S3. Expression and phosphorylation levels of Akt, JNK and ERK proteins in 3T3-L1 adipocytes with insulin stimulation. The protein levels were measured by Western blot using 3T3-L1 adipocytes stimulated with insulin (20nM for 10 min.). Quantitative data are normalized to internal GAPDH and presented as fold changes under each condition compared with adipocytes transfected with PCMV (n=3 to 4 for each condition). * $p < 0.05$

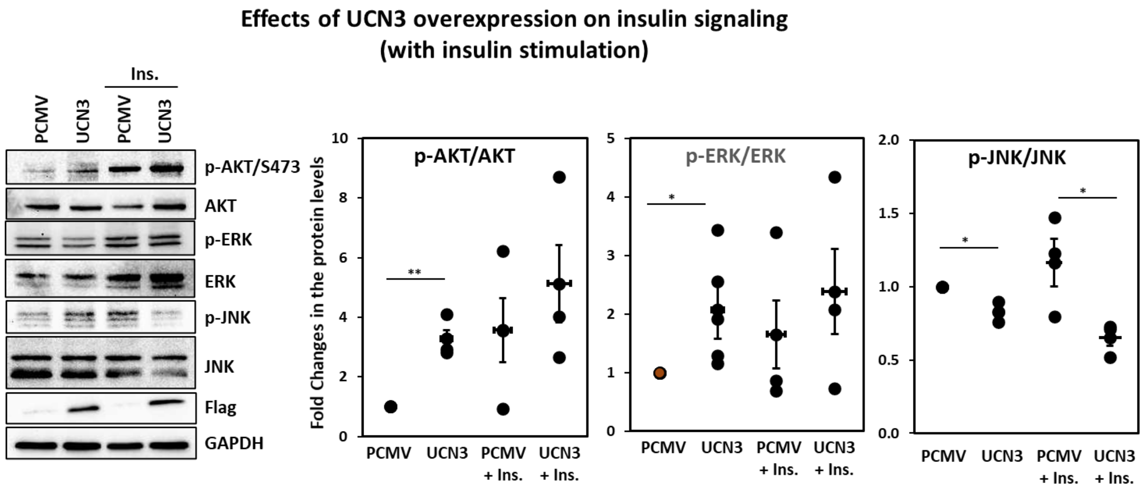

Figure S3

Figure S4. Effects of UCN3 overexpression on the expression levels of HSPs in 3T3-L1 adipocytes treated with macrophage culture medium (MaCM). mRNA expression levels of HSP90, GRP78, HSP72 and HSP60 were measured by RT-PCR in 3T3-L1 adipocytes differentiated for 8 days (d8) with MaCM transfected with UCN3 and PCMV plasmid vectors. GAPDH was used as an internal control for normalization and data are presented as fold changes in differentiated adipocytes compared with adipocytes transfected with PCMV from independent experiments (n=4). * $p < 0.05$

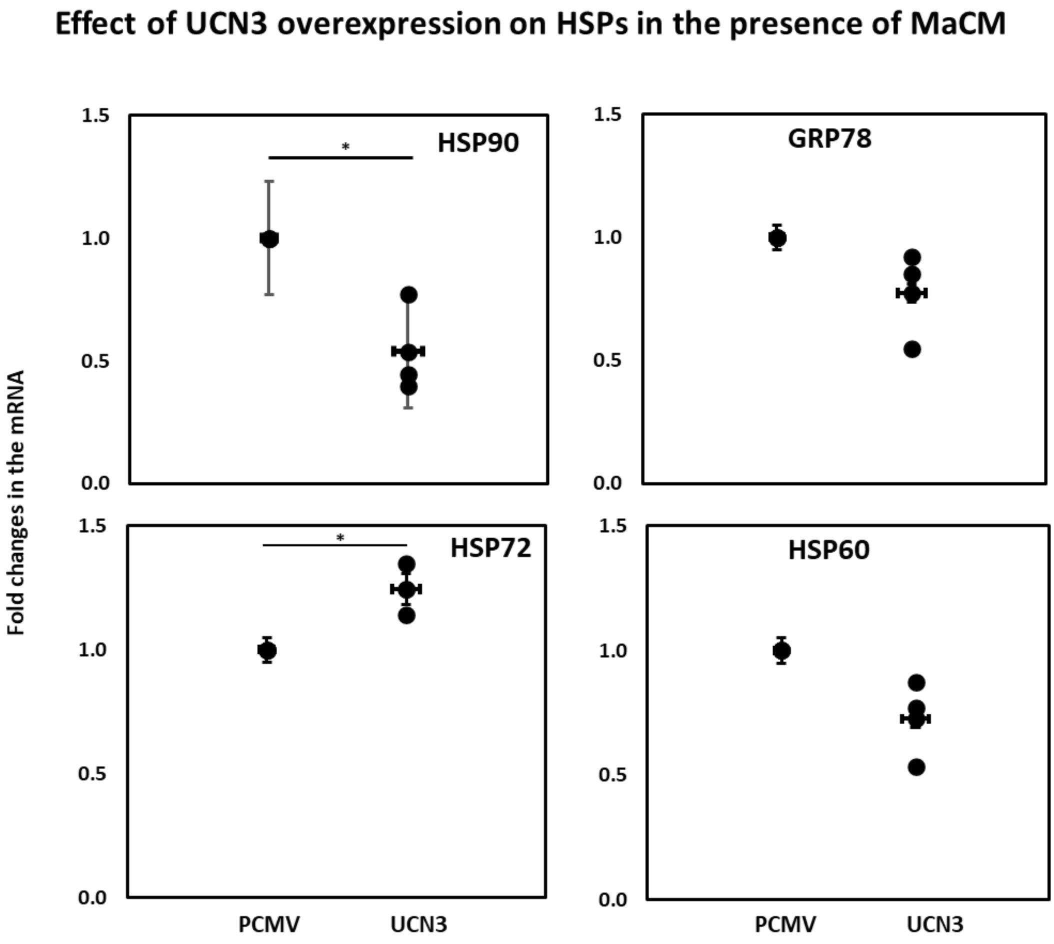

Figure S4

Figure S5. Expression levels of ER stress and apoptotic markers during the differentiation of 3T3-L1 preadipocytes with a macrophage culture medium (MaCM). mRNA expression levels of CHOP, PERK, ATF6 and IRE1α were measured by RT-PCR in 3T3-L1 adipocytes differentiated for 8 days (d8) in the presence of MaCM and transfected with UCN3 or PCMV plasmid vectors. GAPDH was used as an internal control for normalization and data are presented as fold changes in differentiated adipocytes compared with adipocytes transfected with PCMV from independent experiments (n=4). * $p < 0.05$, ** $p < 0.01$.

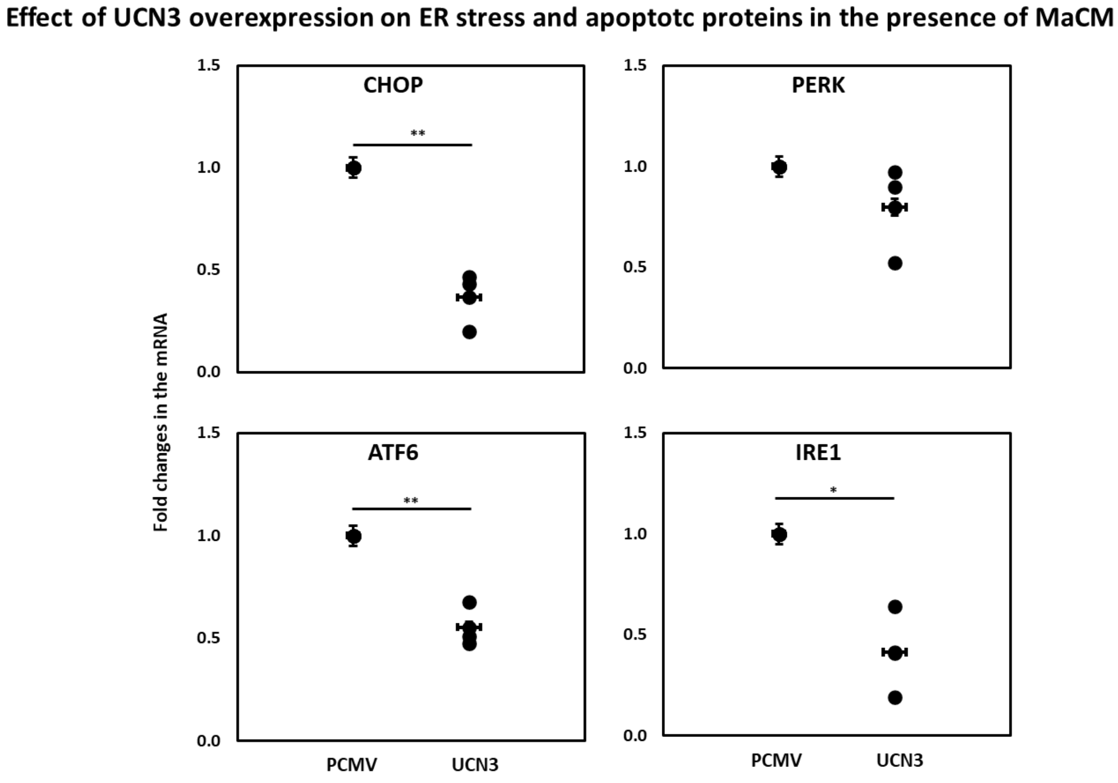

Figure S5

Figure S6. UCN3 overexpression affects IL6 and TNFα expression levels in 3T3-L1 cells. mRNA expression levels of IL6 and TNFα were measured by quantitative real-time PCR in 3T3-L1 adipocytes differentiated for 8 days (d8). GAPDH was used as an internal control for normalization and data are presented as fold changes in adipocytes overexpressing UCN3 compared with adipocytes transfected with PCMV (n=4).

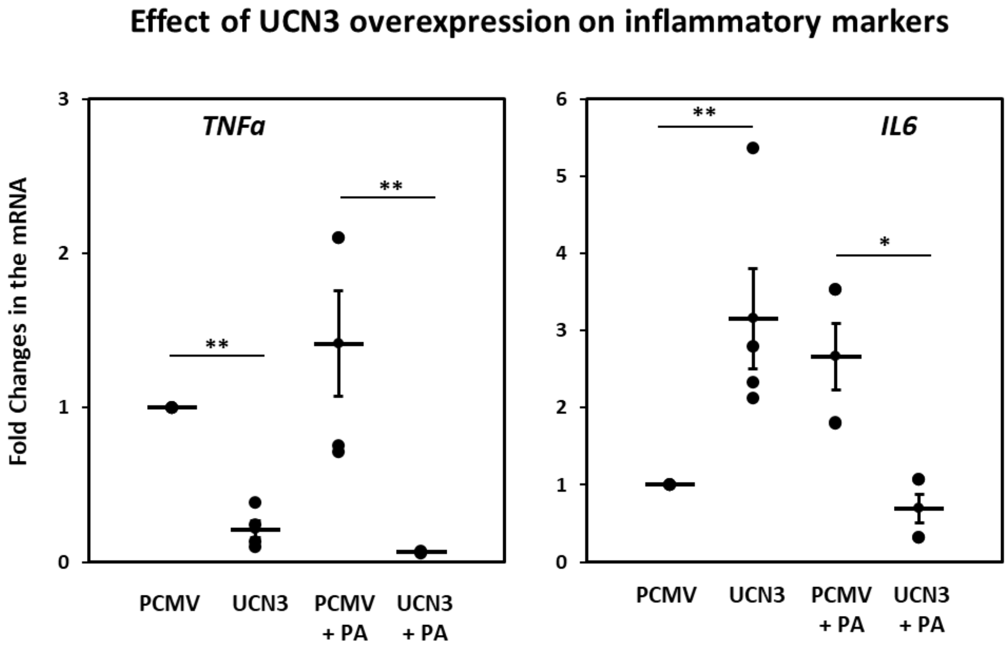

Figure S6

Figure S7. Correlation analysis. HSP60 and GRP78 circulating levels in plasma from 144 nondiabetic adult human with various BMI (25.0 – 39.9 kg/m2), were correlated with UCN3 circulating levels. Protein levels were assessed using ELISA kits (as detailed in [1], [2], and [3], respectively for HSP60, GRP78, and UCN3). Correlations were assessed using Spearman's rank correlation coefficient.

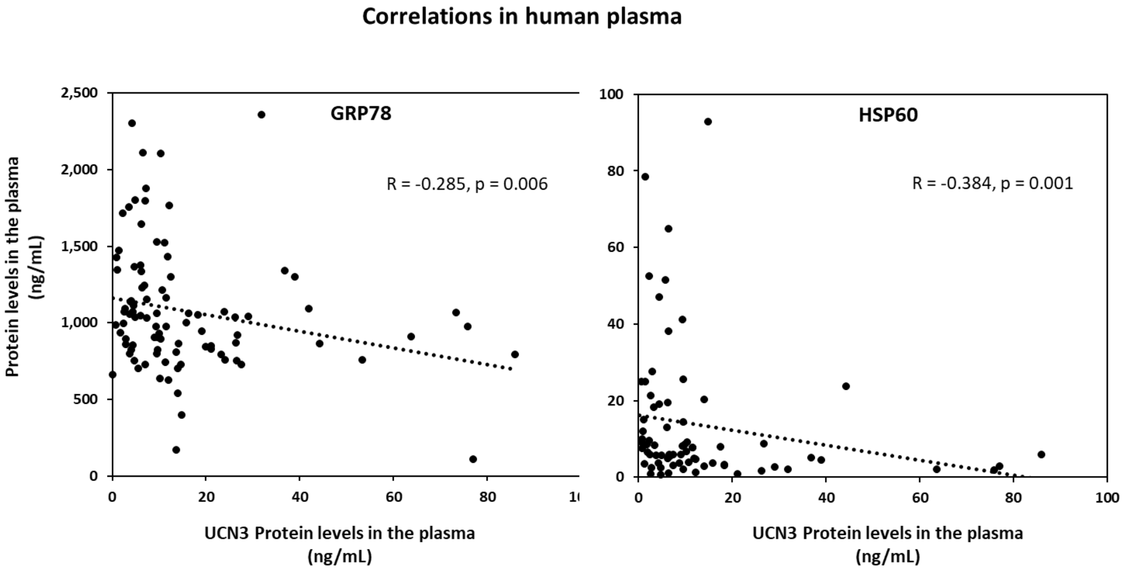

Figure S7

Extra references

1 Khadir, A. *et al.* Physical Exercise Enhanced Heat Shock Protein 60 Expression and Attenuated Inflammation in the Adipose Tissue of Human Diabetic Obese. *Front Endocrinol (Lausanne)* **9**, 16, doi:10.3389/fendo.2018.00016 (2018).

2 Khadir, A. *et al.* Physical exercise alleviates ER stress in obese humans through reduction in the expression and release of GRP78 chaperone. *Metabolism* **65**, 1409-1420, doi:10.1016/j.metabol.2016.06.004 (2016).

3 Kavalakatt, S. *et al.* Urocortin 3 Levels Are Impaired in Overweight Humans With and Without Type 2 Diabetes and Modulated by Exercise. *Frontiers in Endocrinology* **10**, doi:10.3389/fendo.2019.00762 (2019).

FIGURE 2

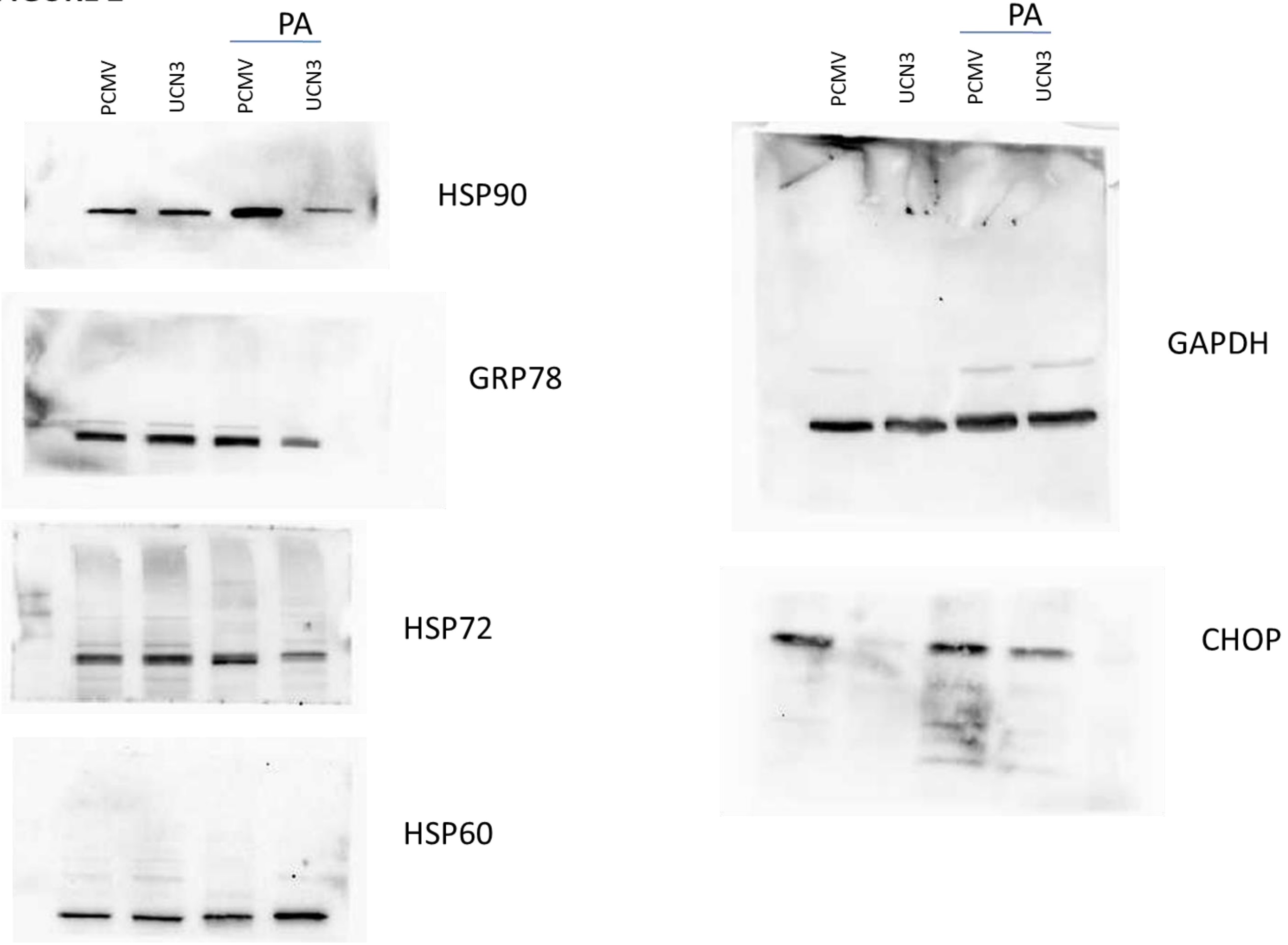
PA
PCMV
UCN3
PCMV
UCN3
HSP90
GRP78
HSP72
HSP60
PA
PCMV
UCN3
PCMV
UCN3
GAPDH
CHOP

FIGURE 5

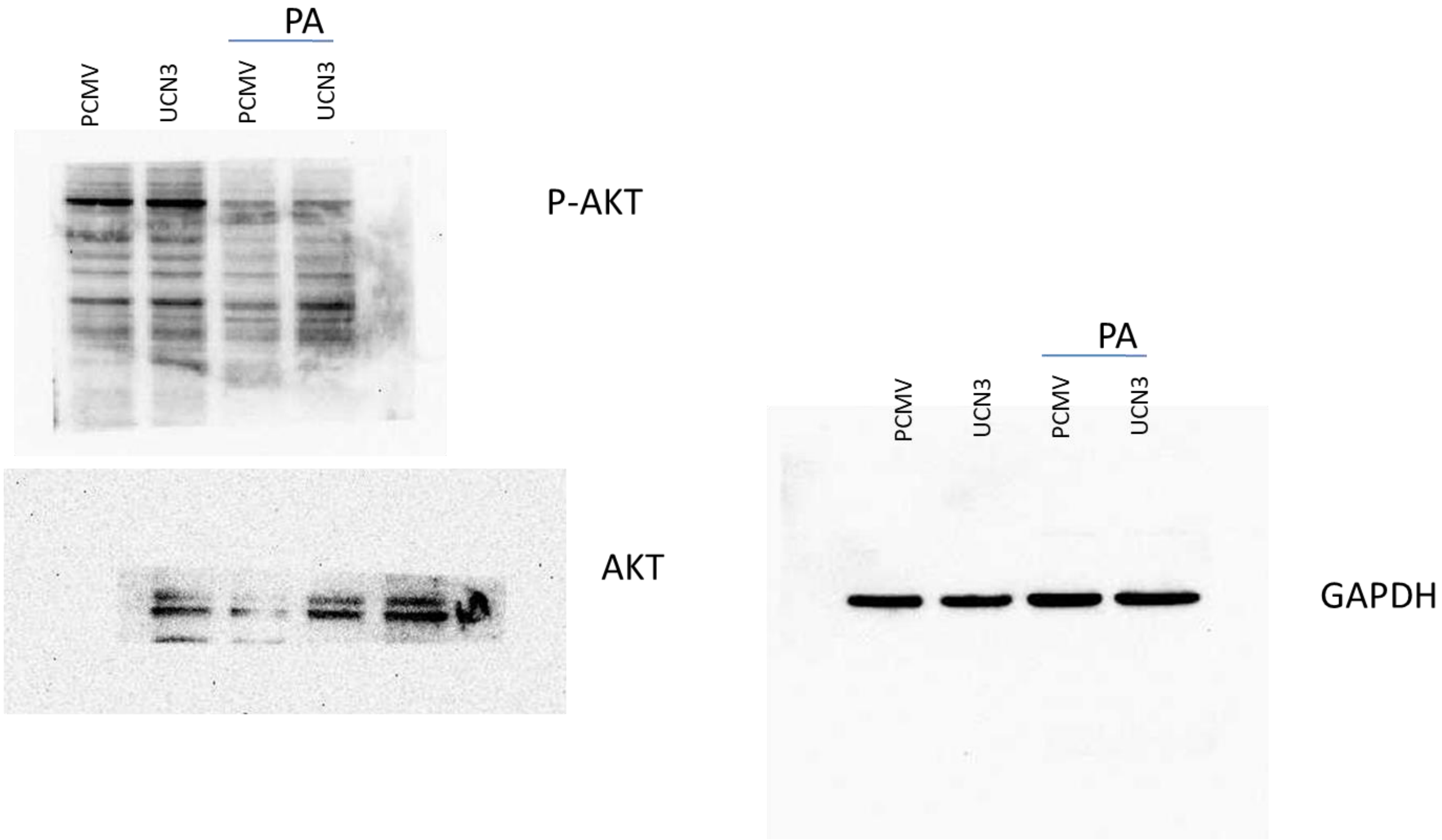

FIGURE 5

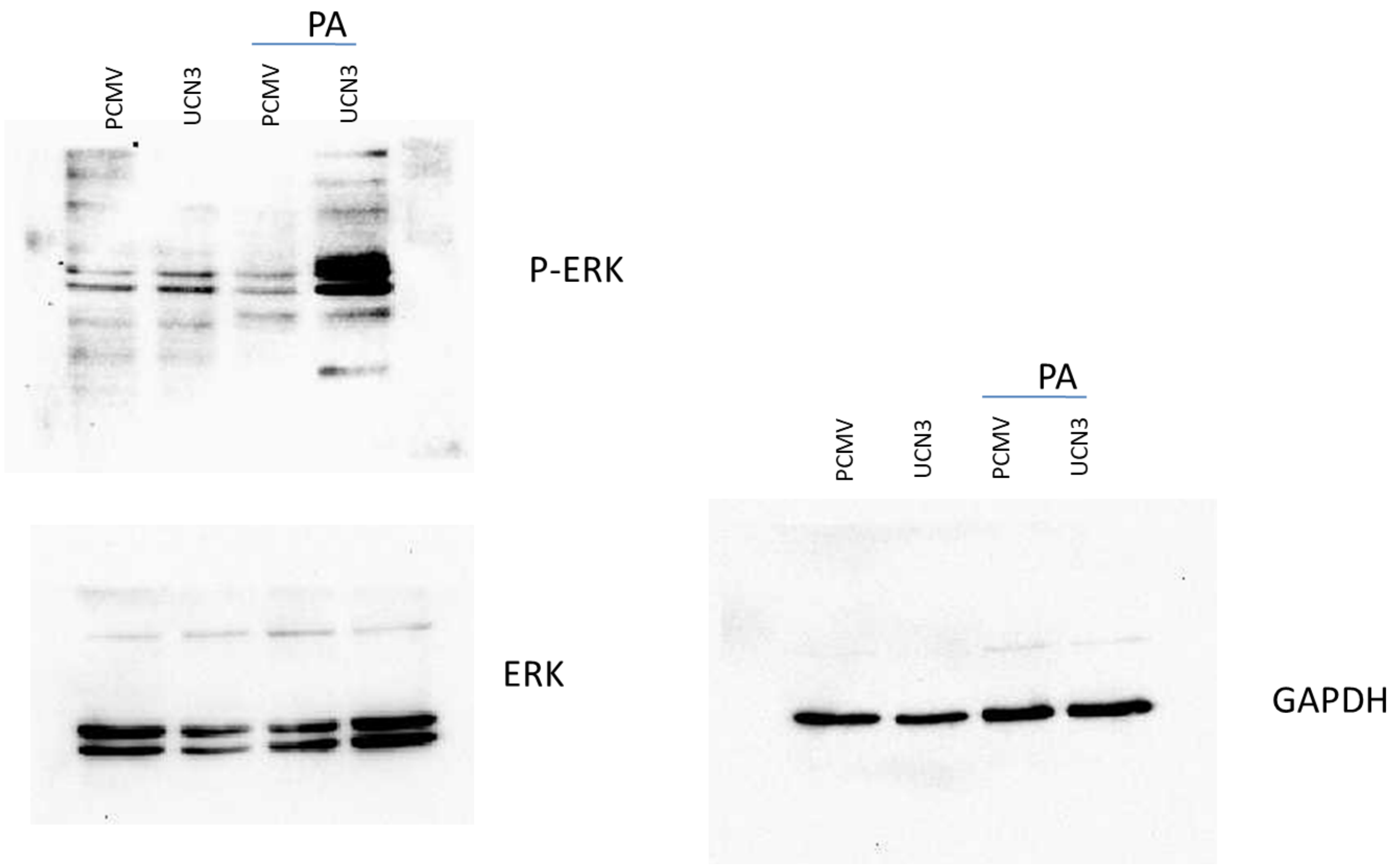

FIGURE 5

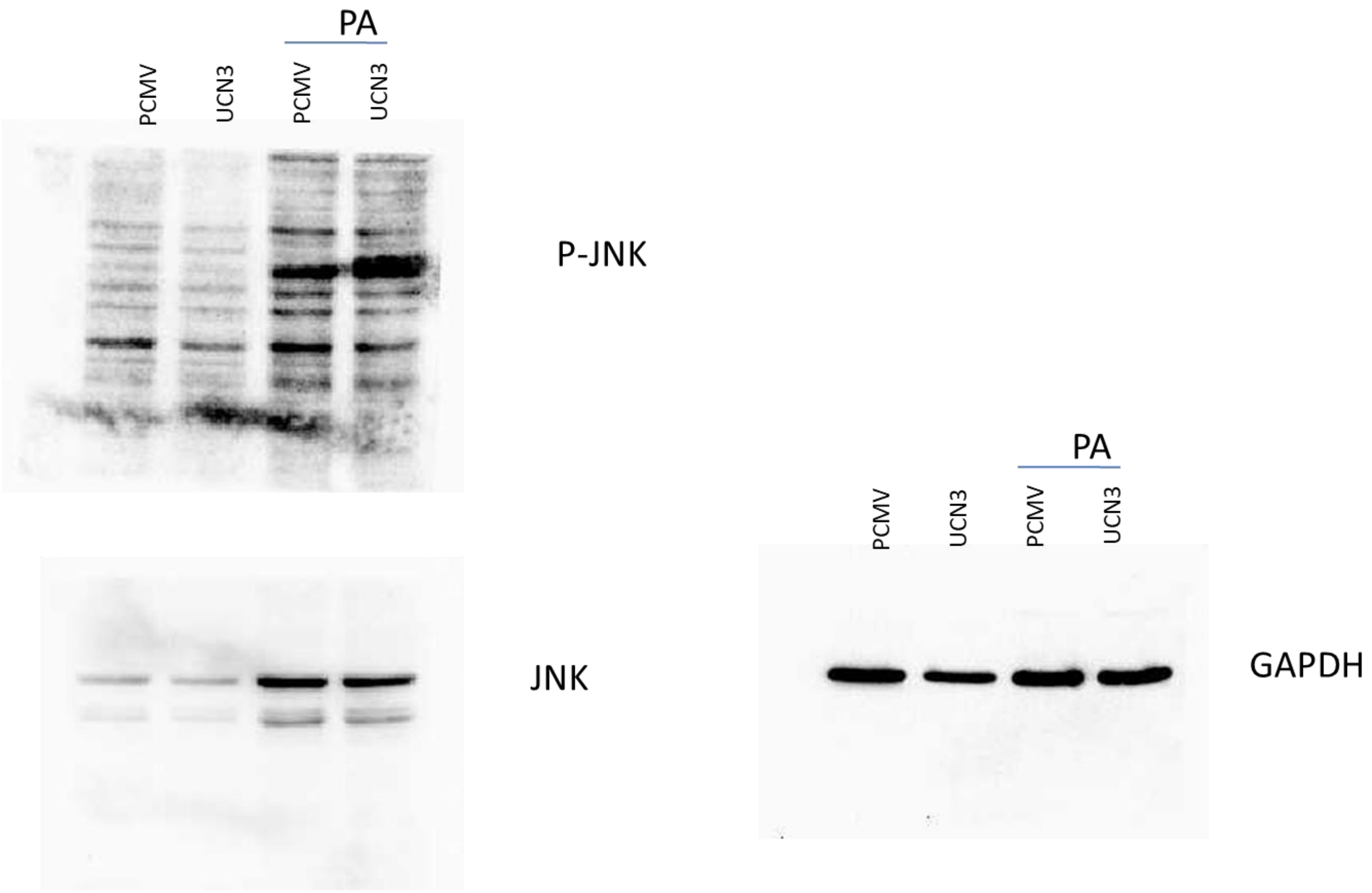

FIGURE S1

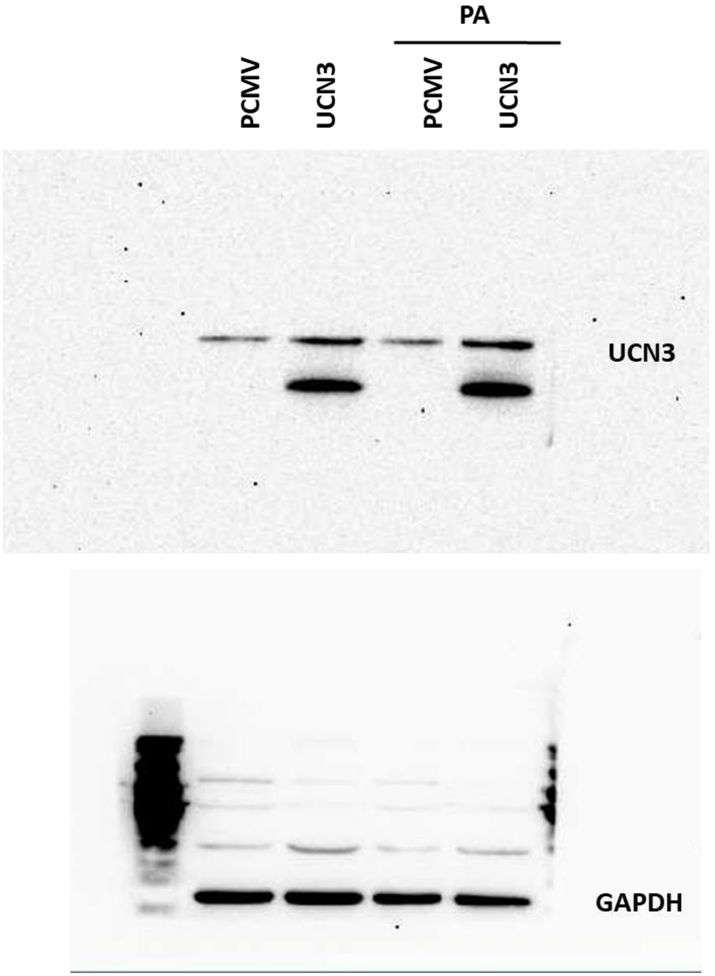

FIGURE S3

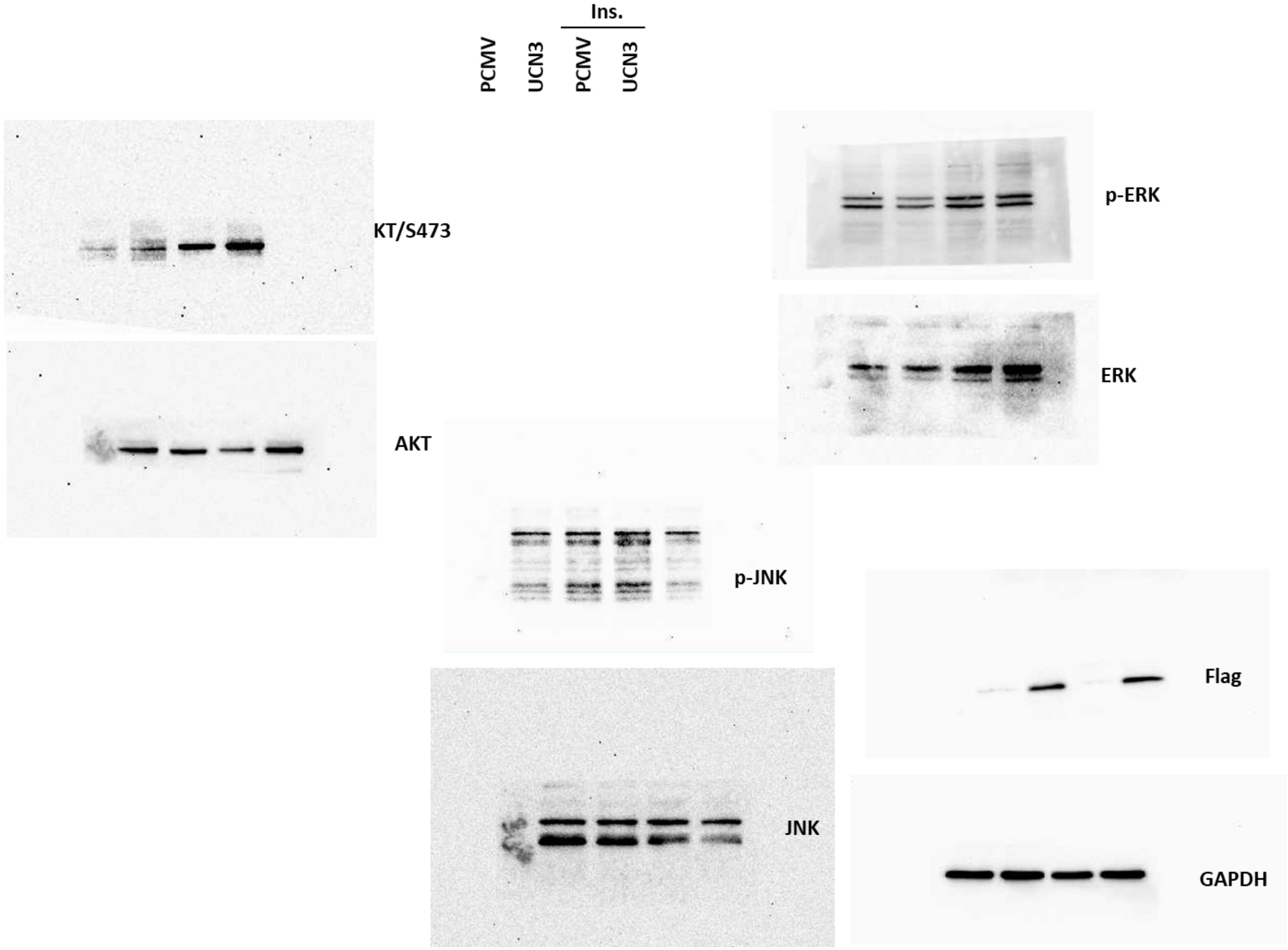
Ins.
PCMV
UCN3
PCMV
UCN3
KT/S473
AKT
p-JNK
JNK
p-ERK
ERK
Flag
GAPDH
